# Repression of liver colorectal metastasis by the serpin Spn4A a naturally occurring inhibitor of the constitutive secretory proprotein convertases

**DOI:** 10.18632/oncotarget.1966

**Published:** 2014-05-13

**Authors:** Fatma Sfaxi, Nathalie Scamuffa, Claude Lalou, Jia Ma, Peter Metrakos, Géraldine Siegfried, Hermann Ragg, Andreas Bikfalvi, Fabien Calvo, Abdel-Majid Khatib

**Affiliations:** ^1^ Université Bordeaux 1, LAMC, Talence, France; ^2^ INSERM, UMR 1029, F-33405 Talence, France; ^3^ Department of Surgery, McGill University, Royal Victoria Hospital, McGill University Health Centre, Montreal, H3A 1A1, QC, Canada; ^4^ Department of Biotechnology, Faculty of Technology, University of Bielefeld, 33501 Bielefeld, Germany; ^5^ Institut de Génétique Moléculaire, INSERM UMRS 940, Université Paris7, Paris, France

**Keywords:** Spn4A, Furin, PC5, PACE4, PC7, mitogenic activity

## Abstract

Liver is the most common site of metastasis from colorectal cancers, and liver of patients with liver colorectal metastasis have abnormal levels of the proprotein convertases (PCs). These proteases are involved in the activation and/or expression of various colon cancer-related mediators, making them promising targets in colorectal liver metastasis therapy. Here, we revealed that the serpin Spn4 from Drosophila melanogaster inhibits the activity of all the PCs found in the constitutive secretory pathway and represses the metastatic potential of the colon cancer cells HT-29 and CT-26. In these cells, Spn4A inhibited the processing of the PCs substrates IGF-1R and PDGF-A that associated their reduced anchorage-independent growth, invasiveness and survival in response to apoptotic agents. In vivo, Spn4A-expressing tumor cells showed repressed subcutaneous tumor development and liver metastases formation in response to their intrasplenic inoculation. In these cells Spn4A induced the expression of molecules with anti-metastatic functions and inhibited expression of pro-tumorigenic molecules. Taken together, our findings identify Spn4A as the only endogenous inhibitor of all the constitutive secretory pathway PCs, which is able to repress the metastatic potential of colon cancer cells. These results suggest the potential use of Spn4A and/or derivates as a useful adduct colorectal liver metastasis prevention.

## INTRODUCTION

By inducing the cleavage and/or expression of various protein precursors, the kexin-like proprotein convertases (PCs) are directly involved in the malignant phenotype of colon cancer cells, and play a key role in colon cancer progression and metastasis [1-7]. To date, a wide range of PC substrates and/or downstream effectors that comprise growth promoting factors and their receptors, adhesion receptors and proteases are known [1-7]. For example, processing by PCs of VEGF-C [3], PDGF A [4], IGF-1 receptor [5] and MT1-MMP [7] was found to be required for induction of tumor cell proliferation and/or invasion. Similarly, induction of liver cytokine and E-selectin by metastatic colon cancer cells, a crucial step for colorectal liver metastasis, was reported to require functional PCs in invading colon cancer cells [6]. The PCs activate their substrates by cleavage at the consensus sequence (K/R)-(X)_n_-(K/R)↓, where n = 0, 2, 4, or 6 and X is any amino acid except Cys [1-7]. Conversion of PC substrates is mediated by one or more of the seven PC family members. These include PC1 and PC2 found within dense core secretory granules that process proteins secreted by the regulated secretory pathway, and Furin, PC4, PC5, PACE4 and PC7 that are involved in processing of protein precursors secreted via the constitutive secretory pathway [1-7]. Altered levels of PCs were reported to be associated with enhanced invasion and proliferation in various tumor cells and tissues, including colon cancer [7, 8]. Conversely, inhibition of PCs activity by the bioengineered PCs inhibitor α_1_-PDX [9] or prodomain of Furin [10] in colon carcinoma cell lines resulted in reduced processing of various PC substrates including colon cancer-related proteins [1-7, 10].

PCs-mediated activation of protein precursors has been found in various organisms and species ranging from Hydra to mammals [11]. However, although the wide distribution and conservation of PCs within the secretory pathway predicts the existence of endogenous inhibitors and/or mechanisms able to regulate their enzymatic functions [2], to date, the naturally occurring PC inhibitors include 7B2 [12], proSAAS [13], CRES [14], PI8 [15] and PAI-1 [16]. 7B2, proSAAS and CRES are only directed against regulated secretory pathway convertases PC2 and PC1/3, with no direct effect on constitutive secretory pathway PCs [11-14]. PAI-1 and PI8 although reported to inhibit Furin activity [17, 18] they also inhibit various proteases. These inhibitions seemed to vary from being marginal to physiologically relevant [15, 16].

Initially, serpins were identified to belong to a superfamily of protease inhibitors that play a regulatory role in blood coagulation, inflammation and complement activation. Mutations in these proteins were found to cause blood-coagulation disorders, cirrhosis, emphysema, and dementia [19-21]. To date, more than 800 genes that encode serpins have been identified in plants, animals, viruses and prokaryotes [19-21]. In Drosophila melanogaster, 29 serpin genes are known, a high number as compared to human. They are involved in maintaining enzymatic homeostasis of various proteases [20, 21]. Previously, the serpin Spn4 gene was suggested as an adaptable defense tool through its ability to encode inhibitors that control the proteolytic function of various serine proteases namely the subtilase family, the chymotrypsin family, and the papain-like cysteine protease family. These include the serpin variants Spn4A, Spn4D, Spn4E and Spn4H [22]. In this study, we show that the serpin Spn4A that contains a Furin cleavage motif in its reactive site loop like α_1_-PDX [23, 24] and reported to inhibit Furin [24] is able to function as an endogenous inhibitor of all the constitutive secretory pathway PCs. We also show that Spn4A represses the malignant and metastatic phenotype of colon cancer cells and blocks the expression and/or activation of several proteins involved in tumor progression and liver colorectal metastasis.

## RESULTS

### Inactivation of cellular Furin, PACE4, PC5A, PC5B and PC7 by Spn4A

**Figure 1:**
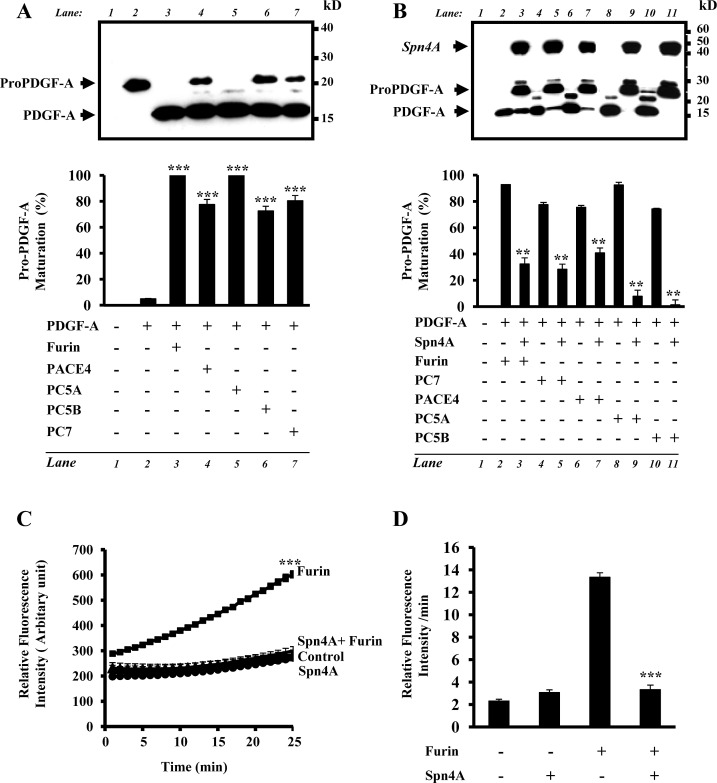
Inhibition of cellular secretory proprotein convertases by Spn4A **(A)**, Western blotting of conditioned media obtained from PC-deficient CHOFD11 cells transfected with either empty vectors (lane 1), empty pIRES2-EGFP vector and pIRES2-EGFP vector containing proPDGF-A (lane 2), or with pIRES2-EGFP vector containing proPDGF-A and pIRES2-EGFP vector that expresses full-length PCs (Lanes 3-7). Note that all indicated PCs are able to rescue proPDGF-A processing in these cells. **(B)**. CHOFD11 cells stably transfected with each of the indicated PCs were transiently transfected with either the empty vectors (lane 1), empty pIRES2-EGFP vector and pIRES2-EGFP vector containing proPDGF-A (lanes 2, 4, 6, 8, 10) or with the pIRES2-EGFP vector containing proPDGF-A and pIRES2-EGFP-V5 vector that expresses full-length Spn4A (Lanes 3, 5, 7, 9, 11). Bars denote the corresponding percentages of pro-PDGF-A processing. **(C)**. Effect of Spn4A on human recombinant Furin was assessed by evaluating Furin (0.2×10^−4^U) ability to digest the fluorogenic peptide pERTKR-MCA at the indicated time points. **(D)** Results shown in the bar graph represent Furin activity after 20 min of incubation. Results are representative of three experiments and data are mean ± S.D performed in triplicate. **P < 0.001. *** *P <* 0.0001.

In contrast to the regulated secretory pathway convertases PC1 and PC2, to date, there is no naturally occurring inhibitor known that regulates the activity of all PCs found in the constitutive secretory pathway Furin, PACE4, PC5A, PC5B and PC7. Interestingly, in Drosophila melanogaster that encodes two Furin homologs [25], and one PC2-like enzyme [26], the use of *in vitro* enzymatic digestion assays revealed that recombinant Drosophila melanogaster **Spn4A** was able to efficiently inactivate Furin *in vitro* [23, 24]. To evaluate the effect of **Spn4A** on enzymatic activity of all constitutive secretory pathway PCs *in vivo*, we first assessed in cells stably expressing Spn4A, the ability of Furin, PACE4, PC5A, PC5B and PC7 to convert human PDGF-A into the mature form. Immunoblotting analysis revealed that media derived from PC-deficient CHOFD11 cells cotransfected with a vector encoding proPDGF-A and control vector, showed only one band (~24 kDa) corresponding to the intact proPDGF-A precursor (Figure 1A, Lane 2). Transfection of CHOFD11 cells that stably express indicated PCs with vector encoding proPDGF-A demonstrated reduction of the precursor protein, and the appearance of the mature form of PDGF-A (Figure 1A, Lanes 3-7). In contrast, when Spn4 was cotransfected together with pro-PDGF-A processing of pro-PDGF-A was inhibited (Figure 1B, lanes 3, 5, 7, 9, 11). These data revealed that Spn4A is able to repress all PCs found in the constitutive secretory pathway when expressed in cells. Using an in vitro enzymatic digestion assay with the fluorogenic peptide pERTKR-MCA as substrate, we confirmed inhibition of PCs by **Spn4A**. The enzymatic activity of recombinant human Furin (0.2×10^−4^U) (Figures 1C, 1D) and of media derived from PC deficient CHOFD11 cells, stably transfected with a vector expressing Furin (FD11/Furin), PACE4 (FD11/PACE4), PC5A (FD11/PC5A), PC5B (FD11/PC5B) or PC7 (FD11/PC7) was inhibited by recombinant Spn4A (Supplementary Figure 1).

### Inhibition of endogenous PCs activity in colon cancer cells by Spn4A

**Figure 2:**
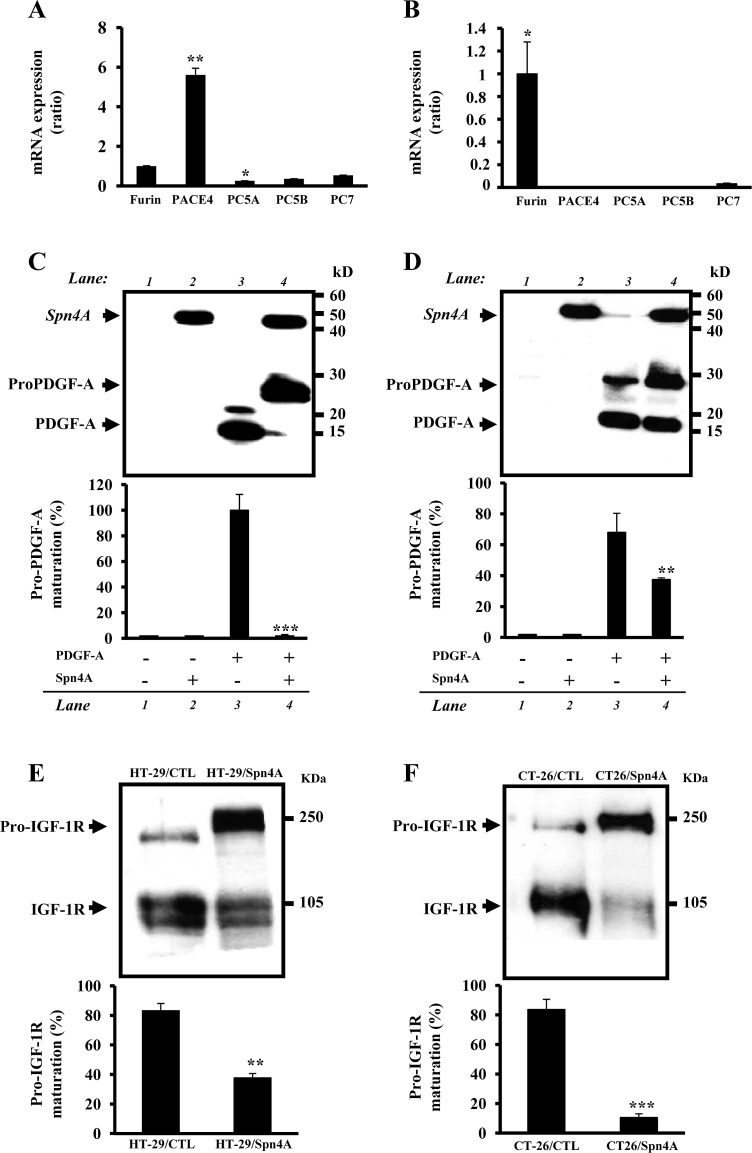
Inhibition of endogenous PCs activity in colon cancer cells by Spn4A **(A-B)**, Using specific primers, the expression of indicated PCs was assessed by Real time-PCR on RNA extracted from HT-29 (A) and CT-26 (B) cells. (**C**–**F**), Endogenous PCs activity was assessed by ability of indicated cells to process proPDGF-A and proIGF-1R using an anti-V5 (**C** and **D**) and anti–IGF-1R antibody (**E** and **F**), respectively. Bars denote the corresponding percentages of pro-PDGF-A and pro-IGF-1R processing. Values are mean ± SD (*n =* 3 per group). *P < 0.05; **P < 0.001. *** *P <* 0.0001.

To assess the possibility that the endogenous proteolytic activity of PCs is inhibited by Spn4A, we analyzed in colon carcinoma cells HT-29 and CT-26 stably transfected with Spn4A ability to mediate maturation of the PC substrates. HT29 cells express all convertases while CT-26 lacks PACE4 and PC5 (Figures 2A, 2B). We next analyzed the processing of two PC substrates, PDGF-A (Figures 2C, 2D) and IGF-1R (Figures 2E, 2F). Both cell lines express IGF-1R while lacking PDGF-A expression. Immunoblotting of media derived from cells transfected with proPDGF-A cDNA revealed that proPDGF-A was significantly processed (Figures 2C lane 3, 2D lane 3). In contrast, when Spn4A is expressed, in addition, in these cells, the maturation of proPDGF-A was inhibited, as demonstrated by the accumulation of its unprocessed form and reduction of its mature form (Figures 2C lane 4, 2D lanes 4). Similarly, using a specific IGF-1R antibody, we found that the processing of the endogenous proIGF-1R β-subunit was also blocked in HT-29 and CT26 cells expressing Spn4A (Figure 2E-F), as evidenced by the accumulation of the precursor form and the reduction of the mature form.

### Inhibition of anchorage-independent growth and proliferation of colon cancer cells by Spn4A

**Figure 3:**
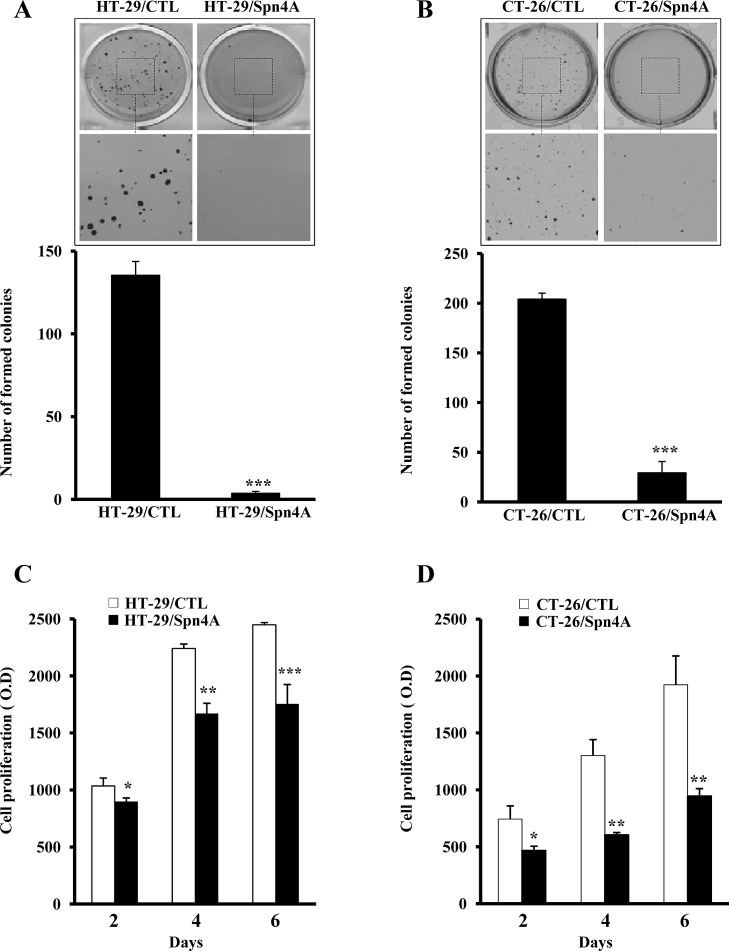
Inhibition of anchorage-independent growth and proliferation of colon cancer cells by Spn4A **(A-B)**, Control (*CTL*) and tumor cells stably expressing Spn4A (Spn4A) were seeded in triplicate in six-well plates in soft agar. After 2 weeks, colonies ≥100 μm in diameter were counted. Results are shown as means ± S.D. of three experiments performed in triplicate. **(C-D)**, Starved control or Spn4A-expressing cells were cultured for 6 days under standard conditions and cell proliferation was assessed using Cell Titer96 non-radioactive cell proliferation assay. Each value results are shown as means ± S.D. of three experiments performed in triplicate. *P < 0.05; **P < 0.001. *** *P <* 0.0001.

We first investigated whether anchorage-independent growth of colon cancer cells was inhibited by Spn4A. Tumor cells expressing Spn4A exhibited more than 80% reduction in their anchorage-independent growth in comparison to control cells (Figures 3A, 3B). Furthermore, control cells but not Spn4 transfected cells grew well in presence of serum (Figures. 3C, 3D).

### Inhibition of PCs activity by Spn4A alters survival and chemosensitivity of colon cancer cells

**Figure 4:**
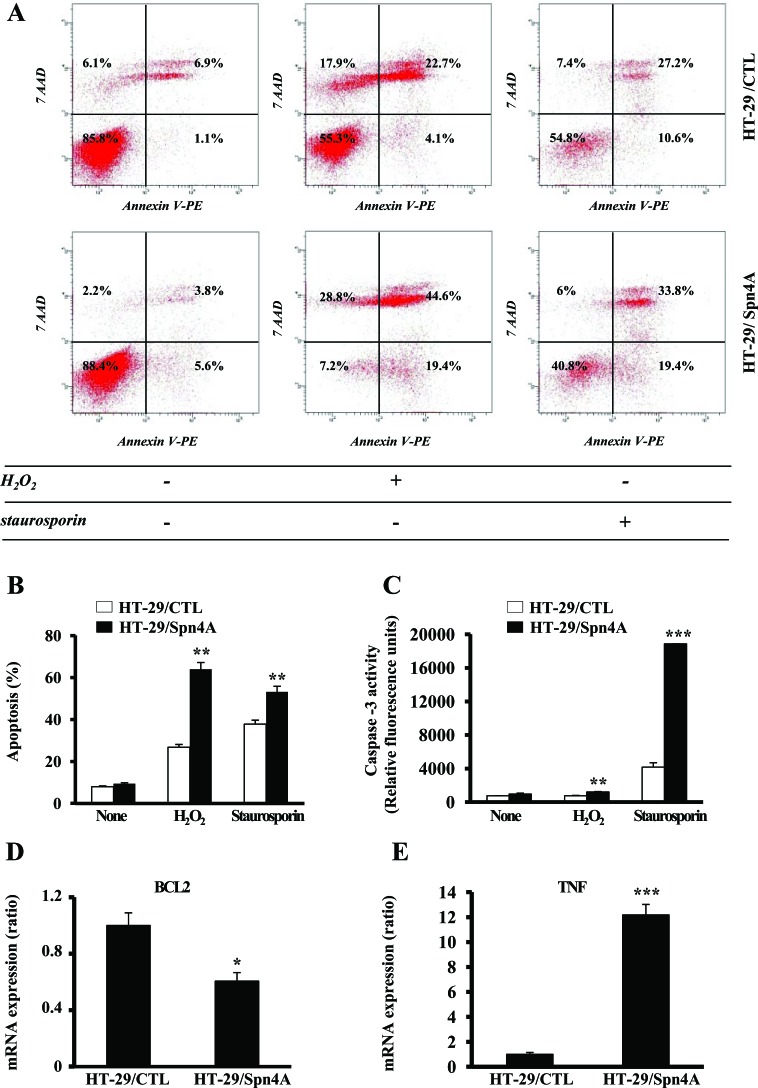
Inhibition of PCs by Spn4A alters tumor cells survival and chemosensitivity **(A)**, FACS scatter plots of HT-29/CTL and HT-29/Spn4A cells incubated for 6 h with H_2_O_2_ (5 mM) or staurosporin (1 μM) and double stained with annexin V and 7AAD. Fluorescence was detected using a fluorescence-activated cell sorter to analyze viable (negative for both dyes; lower left), early apoptotic (Annexin+/7AAD−, lower right), necrotic cells (Annexin-/7AAD+, upper left), and late apoptotic (Annexin+/7AAD+, upper right). **(B)** Percentages of apoptotic cells, under these conditions are indicated. **(C)**, tumor cells were incubated for 6 h with H_2_O_2_ (5 mM) or staurosporin (1 μM) and caspase-3 activity was evaluated using the Caspase-3 Fluorescence assay Kit. Note that H_2_O_2_ and staurosporin caused an increased in the percentage of apoptotic cells that are associated with increased caspase-3 activity. This effect was exacerbated in cells-expressing Spn4A. Data shown represents the mean±SD from at least three independent experiments. **(D-E)**, Data derived from analysis of total RNA analyzed by RT^2^ Profiler PCR array PAHS-502C for oncogenes and tumor suppressor genes that contains probes for *BCL2* and *TNF* genes. For each well, the results are expressed relative to control cells transfected with empty vector which was assigned a value of 1. Values are shown as means ± S.D. *P < 0.05; **P < 0.001. *** *P <* 0.0001.

We then investigated the effect of Spn4 on apoptosis by flow cytometry in colon cancer cells HT-29 under normal conditions, and after treatment with H_2_O_2_ (5 mM) or staurosporin (1 μM) (Figure 4A). Using annexin V and 7AAD as markers, flow cytometric analysis, identified four cell populations: viable (negative for both dyes), early apoptotic (Annexin+/7AAD−), necrotic (Annexin-/7AAD+), and late apoptotic cells (Annexin+/7AAD+). The percentages of these populations under normal condition or after treatment with H_2_O_2_ or staurosporin, are shown in Figure 4B. As illustrated, incubation of control cells with H_2_O_2_ or staurosporin for 6h caused an increase in apoptotic cells which was associated with increased Caspase-3 activity (Figure 4C). This effect was exacerbated in tumor cells-expressing Spn4A, indicating a higher chemosensitivity. Similar results were obtained with the colon carcinoma cells CT-26 (Supplementary Figure 2). Real-time PCR array revealed a downregulated expression of the anti-apoptotic gene *BCL2* in Spn4A-expressing tumor cells and an increased expression of the pro-apoptotic gene *TNF* (Figure 4E, Supplementary Table 1).

### Tumorigenicity of Spn4A-expressing colon cancer cells

**Figure 5:**
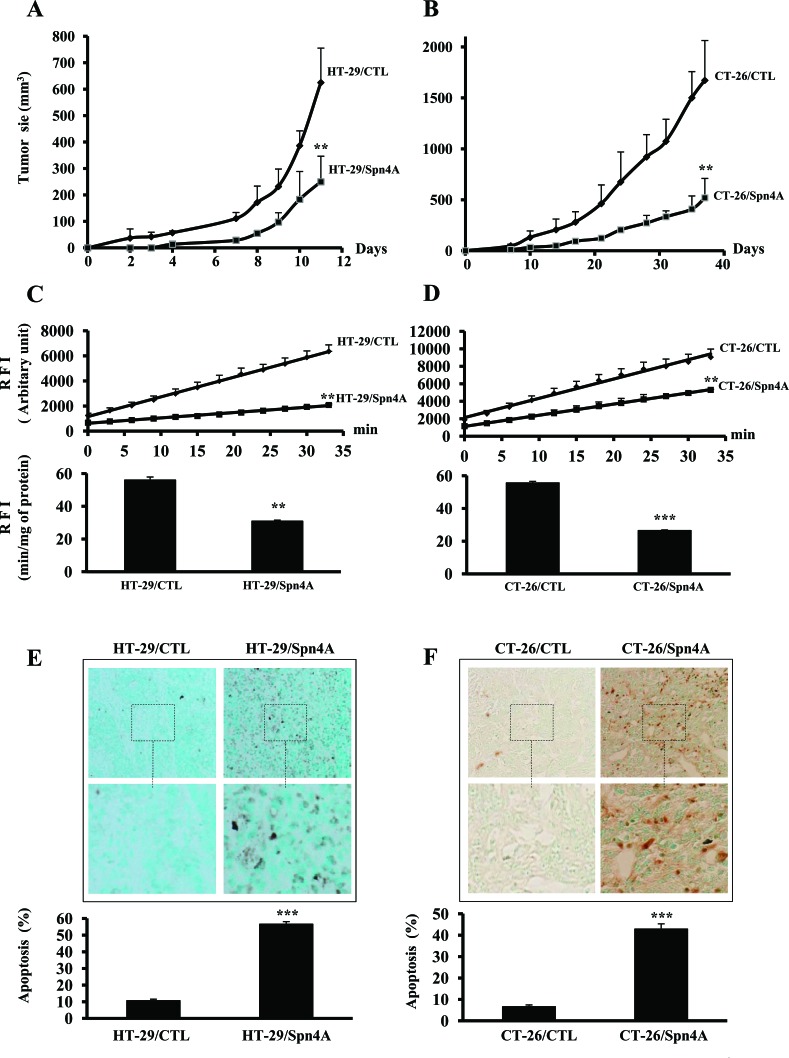
Inhibition of tumor growth by Spn4A is associated with reduced PCs activity and induced apoptosis in developed tumors **(A-B)**, Control (*CTL*) and colon tumor cells stably expressing Spn4A (Spn4A) (1×10^6^) were injected subcutaneously into mice. The animals were monitored for tumor formation every 2-3 days. Results are representative of three experiments. Values are mean ± SD (*n =* 6 per group). *P < 0.05; **P < 0.001. **(C-D)**, Subcutaneously developed tumors were removed and lysed in lysis buffer. Protein extracts were incubated with pERTKR-MCA. Substrate cleavage was evaluated as raw fluorescence intensity (RFI) at indicated time periods. Results shown in the bar graph represent PCs activity after 2 hours of incubation. **(E-F)**, Developed tumors derived from control and Spn4A-expressing tumor cells were analyzed for apoptosis using TumorTACS™ *in situ* apoptosis detection Kit. The number of intra-tumoral apoptotic cells was counted in ten different fields for each tumor. Percentages of apoptotic populations are shown. Data are presented as mean ± SD. ***P < 0.0001. Original magnification × 360.

To assess the effect of PCs inhibition by Spn4A on tumorigenicity of colon cancer cells, four groups of nude mice (n=6) were subcutaneously inoculated with HT-29/CTL, CT-26/CTL, HT-29/Spn4A or CT-26/Spn4A cells. Tumors size was measured at various intervals, and data are summarized in Figures 5A, 5B. Animals injected with Spn4A-expressing tumor cells exhibited a delay in tumor appearance (day 7 for HT-29/Spn4A as compared with day 2 for HT-29/CTL and day 12 for CT-26/Spn4A, as compared with day 5 for CT-26/CTL). Tumor volume, measured at the end of the experiment, was significantly smaller for HT-29/Spn4A and CT-26/Spn4A-derived tumors than for control tumors. In agreement with these finding, the expression of several genes associated with tumor initiation and/or progression including *FOXD3, MEN1,* and *S100a4* was upregulated in Spn4A-expressing cells. However, the expression of other genes involved in tumorigenesis such as *c-REL and JUNB* was down regulated in these cells (Supplementary Figure 3 and Supplementary Table 1).

### Reduced PCs activity in colon cancer cells expressing Spn4A-derived tumors

To evaluate the effect of Spn4A on PCs activity in tumors derived from colon cancer cells, control and Spn4A-expressing cells were subcutaneously injected in mice. Tumors were removed 2-4 weeks later. PCs activity was analyzed by assessing the ability of tumor-derived protein extracts to cleave pERTKR-MCA. The results in Figures 5C and 5D revealed that the extent of substrate cleavage by protein extracts of HT-29/CTL- or CT-26/CTL-derived tumors was higher than that of HT-29/Spn4A- or CT-26/Spn4A-derived tumors. A significant reduction of up to 50% of total PCs activity in tumor cells expressing Spn4A-derived tumors was observed.

### Immunohistochemical analysis of tumor apoptosis

Apoptotic cells in control and Spn4A-expressing cells-derived tumors were analyzed using TumorTACS™ *in situ* apoptosis detection assay. A marked increase (>5-fold) in apoptotic cells in HT-29/Spn4A- and CT-26/Spn4A cell-derived tumors was observed, as revealed by the increased dark brown precipitate within the cells (Figures 5E, 5F).

### Effect of PCs inhibition by Spn4A on migration and invasion of colon cancer cells

**Figure 6:**
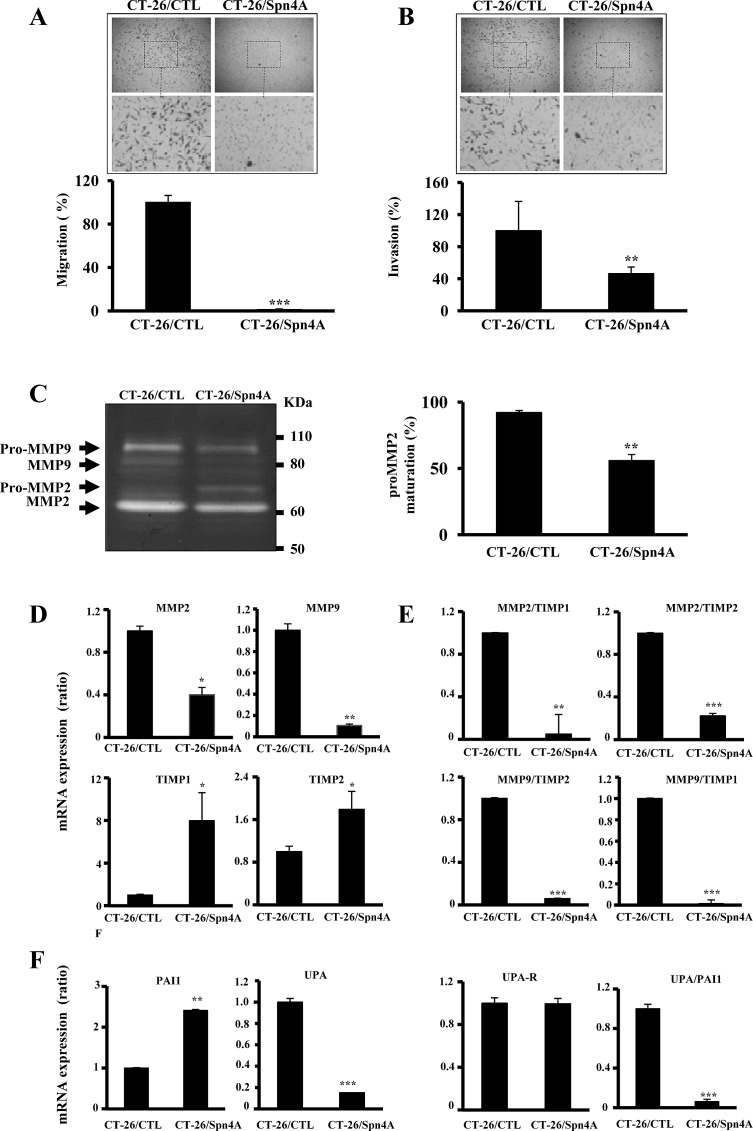
Effect of Spn4A on tumor cell migration and invasion **(A-B)**, Control tumor cells and cells expressing Spn4A were incubated in a microchemotaxis chamber alone **(A)** or pre-coated with collagen IV **(B)**, for cell migration and invasion, respectively. Three independents experiments were done (*n* = 3) and the results are represented as the percentage of migrating and invading cells. **(C)**, Serum-free media derived from the indicated cells were analyzed for gelatinase enzymatic activity. The corresponding percentage of MMP-2 cleavage calculated from the ratio of band intensities of MMP-2/(proMMP-2+MMP-2) is indicated. Note that Spn4A expression inhibited the activity of MMP-2 and MMP-9 and reduced processing of pro-MMP-2. **(D-F)**, Real-time PCR analysis was performed using specific primers for MMP-2, MMP-9, TIMP-1 and TIMP-2, uPA, uPAR, PAI-1 or mB2m. Expression of mB2m that was evaluated in each sample was used as endogenous control. The indicated ratios were deduced from data obtained in **(D)** and **(F)**. Results shown are representative of 3-4 experiments. Data are mean ± SD (*n*=3 per group). *P < 0.05; **P < 0.001. *** *P <* 0.0001.

To evaluate whether inhibition of PCs in tumor cells can affect cell migration and invasion, cells were incubated for 24 hours in a microchemotaxis chamber alone (Figures 6A), or in a chamber pre-coated with collagen IV (Figure 6B). Expression of Spn4A in these cells clearly decreased their ability to migrate and to invade. Furthermore, gelatinase activity in 48h-conditionned serum free media revealed that media derived from control tumor cells presented high MMP-2 and MMP-9 activity. Expression of Spn4A in these cells resulted in decreased MMP-2 and MMP-9 activity (Figure 6C). In Spn4A-expressing cells, processing of MMP-2 was also reduced, as revealed by the accumulation of pro-MMP-2 (Figure 6C). We then analyzed mRNA expression of these MMPs and of their naturally occurring inhibitors TIMP-1 and TIMP-2, using real-time PCR. We found that while expression of MMP-2 and MMP-9 was reduced in Spn4A-expressing tumor cells, mRNA expression levels of TIMP-1 and TIMP-2 was significantly increased (Figure 6D). Evaluation of MMPs/TIMPs ratios indicated that compared to control cells, these ratios were significantly decreased in Spn4A-expressing tumor cells (Figure 6E). Similarly, since the uPA/uPAR/PAI-1 system is also involved in cell invasion, we analyzed the effect of PCs inhibition by Spn4A on expression levels of these molecules. Figure 6F shows that in Spn4A-expressing cells, the level of uPA was reduced; PAI-1 in contrast was increased, whereas the level of uPAR mRNA remained unchanged. Evaluation of uPA/PAI-1 ratios indicated that, compared to control cells, these ratios were decreased in Spn4A-expressing tumor cells. Similarly, further analysis revealed that the expression of other genes involved in extracellular matrix degradation (ECM) including *MMP11, MMP12, MMP13, MMP15* and *ADAMTS1* was reduced. *MMP-8* expression in contrast was increased (Supplementary Figure 4 and Table 1), suggesting the contribution of this global effect of PCs inhibition by Spn4A on cell migration and invasion.

### PCs blockade by Spn4A prevents colorectal liver metastasis

To evaluate the effect of PCs inhibition by Spn4A on the ability of colon cancer cells to colonize the liver, HT-29/CTL, CT-26/CTL, HT-29/Spn4A, and CT-26/Spn4A cells, were injected in mice through the intrasplenic/portal route. At 2 weeks after injection of CT-26/CTL and CT-26/Spn4A cells, and 4 weeks after injection of HT-29/CTL and HT-29/Spn4A cells, livers were removed and the number of metastases was determined (Figures 7A, 7B). In mice inoculated with tumor cells, the number of hepatic metastases was reduced by up to 75% (P < 0.001) in HT-29/Spn4A- and CT-26/Spn4A-injected mice relative to control animals (Mann-Whitney test, Figure 7B). Similarly, PCR Array analysis of ECM proteins and adhesion molecules, known to be involved in invasion and metastasis revealed their reduced expression in Spn4A-expressing cells. These include *COL6A1, CTNND2, TNC, HAS1, ITGA4, ICAM1, NCAM1, VCAM1, CLEC3B* and *LAMA3* (Supplementary Figures 5, 6 and Table 1).

### PC activity in colon cancer cells-derived hepatic metastases

To evaluate the effect of PCs activity in metastatic livers, tumor cells were injected in mice through the intrasplenic/portal route. After 2 or 4 weeks, the livers were removed and lysed. PCs activity was analyzed by assessing their ability to digest pERTKR-MCA. The results shown in Figure 7C, revealed that PCs activity in HT-29/Spn4A- and CT-26/Spn4A-derived metastases were reduced as compared to HT-29/CTL- and CT-26/CTL-derived metastatic livers.

**Figure:**
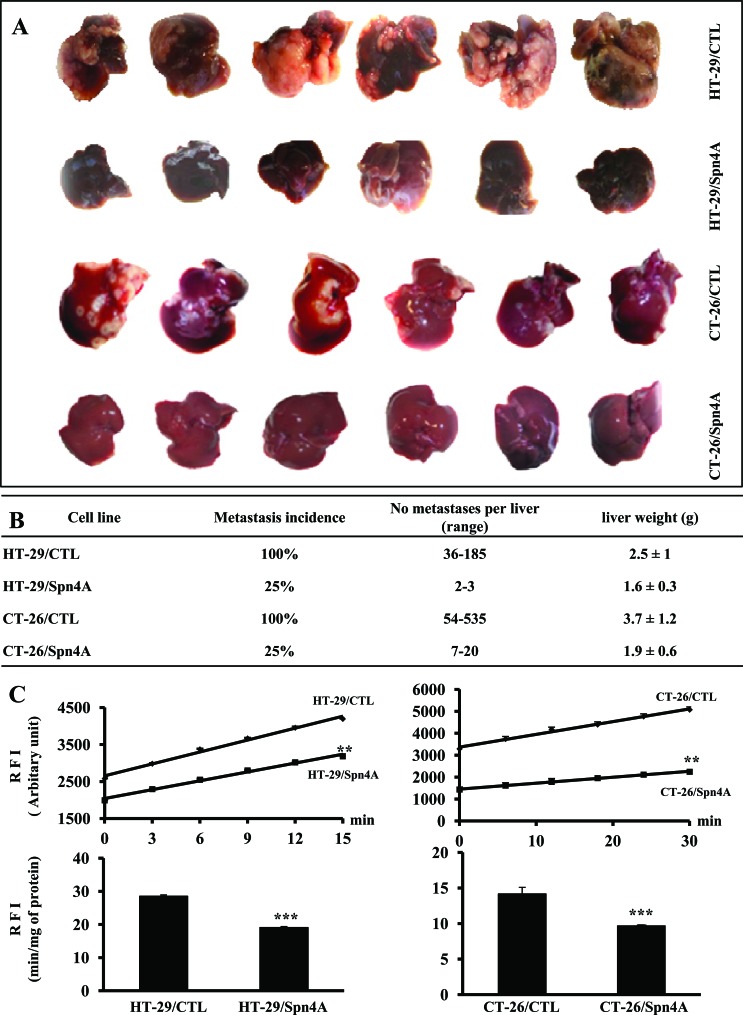
Repression of experimental liver metastasis by Spn4A (**A**), Experimental liver metastases were generated by intrasplenic/portal injection of control and Spn4A-expressiong tumor cells. Liver metastases were enumerated 2 weeks after injection of CT-26 or CT-26/Spn4A cells and 4 weeks after injection of HT-29 or HT-29/Spn4A cells. (B), Data summary of metastasis incidence and prevalence weight. Shown are results of one representative experiment of three performed (n =6 per group, Mann-Whitney test). (**C**), Developed liver tumors derived from control and Spn4A expressing cells­-inoculated mice were analyzed for PCs activity, by assessing their ability to digest pERTKR-MCA. Results shown in the bar graph represent PCs activity after 20 min of incubation. Data are presented as mean ± SD. **P < 0.001. *** *P <* 0.0001.

## DISCUSSION

Dysregulation of PCs expression and/or activity was found to be frequently implicated in a variety of human tumors, including colon cancer [7, 8] and has been linked to aggressive behavior, as revealed by several tumor model systems [1-6]. The clinical relevance of these proteases in colon cancer is substantiated by their ability to proteolytically activate a wide range of proteins responsible for the malignant and metastatic phenotype of colon cancer cells. These include various proteases, growth factors and their receptors and adhesion molecules [1-7]. Thereby, the selective inhibition of PCs activity in cancer cells was recently suggested as a potential important strategy for the treatment of various malignancies, including colon cancer [1, 2, 8, 10].

Previously, the conserved biological functions of PCs in various species predicted the potential existence of endogenous inhibitor(s) or modulator(s) able to regulate PCs proteolytic activity [2]. Similarly, altered expression and/or activity of these proteases under pathological conditions, including neoplasia, reinforced the idea of potential defects in the expression and/or activity of such likely endogenous PC modulator(s). Previously, the naturally-occurring Drosophila melanogaster serpin Spn4A was identified to inhibit Furin in vitro and in insect cells [9]. In this study, we demonstrated that Spn4A was able to efficiently inactivate all the PCs found in the constitutive secretory pathway and to repress growth, survival, invasiveness and metastatic potential of colon cancer cells. Although Spn4A belongs to a large superfamily of protease inhibitors conserved in various species, the members of which operate as a suicide substrate inhibitors by binding covalently to their target proteases [19-24, 27, 28], so far no functional homolog of Spn4A is identified in human.

The high number of serpin genes found in the Drosophila melanogaster genome, as compared to those in human genome seems to correlate with a correspondingly high number of serine proteases [19-24, 27, 28]. This serpins diversity, may contributed to Spn4A presence in Drosophila melanogaster but not human genome. Spn4A is the unique serpin in melanogaster with a carboxyl-terminal His-Asp-Glu-Leu sequence, a functional variant of the KDEL motif that directs proteins to the endoplasmic reticulum [19-24, 27, 28]. This suggests that Spn4A may function within the secretory pathway. In addition, analysis of Spn4A amino acid sequence revealed the presence of the consensus Furin cleavage site RRKR, in its reactive site loop (RSL), suggesting its ability to inhibit PCs as reported for α1-PDX, a bioengineered serpin inhibitor [9]. Indeed, earlier reports revealed that α1-PDX is a general PCs inhibitor able to block the processing of various PC substrates, including those known to be responsible for the acquisition of the malignant phenotype such as IGF-1R, PDGF, and VEGF-C [2-6]. Expression of α1-PDX in various tumor cells resulted in their reduced ability to mediate tumor progression and metastasis [2-7]. Interestingly, Spn4A was reported to be 50-fold more potent than α1-PDX while inhibiting Furin *in vitro* (Ki: 13 pM) [9], highlighting its possible potent inhibitory activity on tumor cells. Indeed, expression of Spn4A in various cells caused the inhibition of Furin-, PC5A-, PC5B-, PACE4- and PC7-mediated proPDGF-A processing (Figure 1). Expression of Spn4A in the colon carcinoma cell lines HT-29 and CT-26 that endogenously produce PCs, blocked the proteolytic cleavage of proPDGF-A and proIGF-1 R (Figure 2). These protein precursors are processed by Furin, PC5A, PC5B, PACE4 or PC7. Processing was found to be required for mediation of their functions in normal and tumor cells [1-7]. Therefore, the effect of Spn4A on HT-29 and CT-26 tumor cells is most likely due to the inhibition of processing of these and probably other substrates associated with the malignant phenotype of colon cancer cells [1-7]. Previously, overexpression of PCs in tumor cells was found to lead to increased cell proliferation and invasion, both *in vitro* and *in vivo* [1] and predicts decreased survival in cancer patients [29]. Our study shows that Spn4A like α1-PDX significantly alters the ability of tumor cells to proliferate and to form colonies in soft agar (Figure 3). In vivo, Spn4A delayed tumor development with significantly decreased size when nude mice were inoculated with colon carcinoma HT-29 or CT-26 cells-expressing Spn4A (Figure 5). Likewise, injection of HT-29/Spn4A and CT-26/Spn4A cells into hepatic circulation inhibited liver metastasis, as compared to controls (Figure 7). Interestingly, the absence of PC5 and PACE4 in CT-26 cells seemed to be associated with their high sensitivity to Spn4A-mediated malignant phenotype inhibition, although these cells are more aggressive as compared to HT-29 cells. Indeed, following their intrasplenic/portal route inoculation; the livers were completely full of metastases within 2 weeks whereas similar results were obtained with HT-29 cells only 4–5 weeks following their inoculation.

The activity of several growth factors and/or their receptors was reported to be required for the mediation of their proliferative and anti-apoptotic function. Indeed, overexpression of IGF-1R was found to induce the growth of tumor cells and to protect cells from apoptosis under a wide variety of proapoptotic inducers. Conversely, when IGF-1R function was blocked by various strategies, tumor cells undergo apoptosis. This results in a dramatic inhibition of tumorigenesis and metastasis [7, 30]. In our model, tumor cells expressing Spn4A exhibited a high chemosensitivity, as revealed by treatment with H_2_O_2_ or staurosporin (Figure 4) and induced apoptosis in HT-29/Spn4A and CT-26/Spn4A cells-derived tumors (Figure 5). This reinforces the importance of PCs in tumor cell survival, probably through activation/expression of various anti-apoptotic mediators such as *BCL2* (Figure 4D). Enhanced apoptosis mediated by Spn4A may be also explained by blockade of an autocrine/paracrine mechanism associated with cell survival. A likely scenario may involve secreted ligands and/or their receptors, which require processing by PCs. Examples, include IGF-1 and IGF-2 that are produced by HT-29 and CT-26 cells [7, 31] and processed by PCs [7]. Since overexpression of Spn4A in these cells inhibited the processing of IGF-1R (Figure 2) and these ligands, this may abrogate their autocrine/paracrine protective effects.

The effect of PCs on cell invasiveness is mediated by activation/expression of molecules involved in cell invasion such as MMPs and urokinase [2, 32]. These molecules degrade basement membranes and the extracellular matrix, which favors tumor cell invasion. In the present study, we found that Spn4A inhibited cell migration and invasion (Figure 6). These alterations were associated with reduced MMP-2 and MMP-9 activity and increased TIMP-1 and TIMP-2 expression. These findings indicate that loss of MMPs activity resulted in increased TIMP-1 and TIMP-2 expression, in addition to the effect of Spn4A on the maturation blockade of MMP-2 (Figure 6). MMP-2 was previously reported to be processed by MT1-MMP that is activated by Furin-like enzymes [7]. Furthermore, MT1-MMP is also involved in extracellular matrix remodeling either directly or through activation of other procollagenases [33]. In addition, PCR Array analysis for various genes involved in ECM degradation and tumor cells migration revealed their altered expression in Spn4A-expressing cells (Supplementary Table-1). These findings indicate that Spn4A inhibited tumor growth and metastasis by repressing PCs activity. This results in the reduced activation/expression of various molecules involved in tumor progression and liver metastasis. Indeed, PCs activity was reduced in HT-29/Spn4A and CT-26/Spn4A-derived subcutaneous tumors and hepatic metastases (Figure 5, Figure 7). In agreement with our results, several studies have shown that the inhibition of Furin led to successful blockade of tumor growth and invasion in many animal cancer models [1, 4, 34]. Additionally, PACE4, PC5, and PC7 inhibition resulted in processing blockade of various PC substrates such as PDGF-A [4] and VEGF-C [3]. Since these are key factors in tumor angiogenesis and progression, development of new PC inhibitors may show some promise. In a phase I trial (FANG vaccine trial), an autologous tumor-based product incorporating a plasmid encoding GM-CSF and a bifunctional short hairpin RNAi (bi-shRNAi) targeting Furin was recently found to be beneficial with 91% success rate in patients with advanced cancer [35]. The FANG vaccine was safe and elicited an immune response in patients, which led to prolonged survival.

Previously, several endogenous protease inhibitors in addition to their cognate enzymes were reported to inhibit Furin activity. These include the ovalbumin-type serpin human proteinase inhibitor-8 (PI8) and plasminogen activator inhibitor 1 (PAI-1) [15, 16]. PI8, containing two instances of the minimal Furin recognition sequence, was shown to inhibit Furin in a rapid, tight binding manner that is characteristic of physiological serpin-proteinase interactions [15]. However, the cytosolic localization of inhibition, inhibitor-8 requires the addition of a signal peptide before it could inhibit Furin in vivo, and even then its ex vivo inhibitory property is yet to be proven. Similarly, based on its inhibitory action on plasminogen activators, PAI-1 is considered to be a major regulator of fibrinolysis. However, PAI-1 inhibits several other serine proteases; this inhibition can be physiologically appropriate or irrelevant, owing to a variable affinity of PAI-1 with its targets [16]. In this context PAI-1 was found to form an SDS-stable complex with the Furin, and inhibits its activity [17]. In addition to their lack of specificity toward Furin, in our knowledge there is no reports that describe PAI and PI8 ability to inhibit all the constitutive secretory pathway PCs. Taken together, our in vitro and in vivo results provide evidence that Spn4 encodes the only effective naturally occurring inhibitor characterized to date that is able to inhibit these PCs which results in an impairment of tumor growth, invasion and metastasis. Spn4A possibly will constitute a starting point for the development of a new class of inhibitors that may be useful adduct for the prevention of colorectal liver metastasis.

## MATERIALS AND METHODS

### Cell culture and transfection

The human HT-29 colon adenocarcinoma cell line, murine CT-26 colon adenocarcinoma cells and PC-deficient CHOFD11 cells were maintained in DMEM, RPMI and DMEM/F12 media, respectively, supplemented with 10% FCS, 100 units/ml penicillin, and 100 μg/ml streptomycin (Invitrogen). Prior cell transfection, Spn4A cDNA was cloned into SacII/EcoRI-digested pIRES2-EGFP-V5 [3, 4]. Cells were stably transfected with empty pIRES2-EGFP-V5 vector (HT-29/CTL, CT-26/CTL and CHOFD11/CTL) or with pIRES2-EGFP-V5 vector containing full-length Spn4A cDNA (HT-29/Spn4A, CT-26/Spn4A and CHOFD11/Spn4A). To generate cells expressing Spn4A, HT-29/Spn4A and CT-26/Spn4A cells were cultured in presence of *Pseudomonas* exotoxin A. This toxin induces cells death only after its cleavage by PCs [36]. The expression of Spn4A-V5 was assessed by immunoblotting and cells were grown in their adequate media supplemented with 200 μg/ml G418. To evaluate the effect of Spn4A on PC substrates, processing by each of individual PC found in the secretary pathway, CHOFD11 cells were stably transfected with Furin, PACE4, PC5A, PC5B or PC7. In other experiments, CHOFD11, HT-29, CT-26, HT-29/Spn4A and CT-26/Spn4A cells were transiently transfected with empty pIRES2-EGFP-V5 vector or with the same vector containing full-length PDGF-A cDNA to study proPDGF-A processing. All transfections were carried out using Lipofectamine reagent (Invitrogen), as recommended by the manufacturer.

### Real-time PCR

Total RNA was extracted using Trizol reagent (Invitrogen) according to the manufacturer's instructions. One μg of total RNA was subjected to cDNA synthesis using the high capacity cDNA reverse transcription kit (Applied Biosystems, Courtaboeuf, France). The relative quantification of specific mRNAs was performed by real-time PCR using the StepOnePlus™ Real-Time PCR System and PCR Master Mix (Applied Biosystems, Courtaboeuf, France) according to the manufacturer's instructions. Briefly, the reaction mixture (20 μl) contained 2 μl of cDNA resulting of 5-fold dilution of RT mixture product, 2 × TaqMan Universal PCR Master Mix, 0.3 μM of the probe and 0.9 μM of the forward and reverse primers (Supplementary Table 2). PCR reactions were performed at 94°C for 15 s and at 60°C for 1 min during 40 cycles. hsB2m and mHPTRT1 transcriptions levels evaluated in each sample, were used as endogenous controls for human and mouse cells, respectively.

### RT^2^Profiler PCR Array

The RT^2^ Profiler PCR Array was used as a method of combining real-time PCR performance with a simultaneous analysis of a panel of genes related to human oncogenes and tumor suppressor genes (Array PAHS-502C) and to human extracellular matrix and adhesion molecules (array PAHS-013C). Preparation and analysis of samples were carried out in accordance with the manufacturer's recommendations (SABiosciences).

### Immunoblotting

Cells were lysed in Triton buffer (50 mmol/l Tris–HCl (pH = 7.4), 250 mmol/l NaCl, 1 mmol/l EDTA (pH = 8), 0.1% Triton) containing protease inhibitors (Roche). Media or lysates were subjected to SDS-polyacrylamide gel electrophoresis and proteins were blotted onto nitrocellulose membranes. The primary antibodies used were anti-IGF-I receptor (Santa Cruz Biotechnology) and anti-V5, for detection of PDGF-A-V5 and Spn4A-V5 (Invitrogen), respectively. Primary antibodies were revealed by horseradish peroxidase-conjugated secondary antibodies (Amersham, Pharmacia Biotech) and Enhanced Chemiluminescence (ECL+Plus, Amersham Pharmacia Biotech) according to the manufacturers' instructions.

### Measurement of proprotein convertases activity

The effect of Spn4A on PCs activity in cells and tissues was assessed by the evaluation of the enzymes ability to digest the universal PC substrate, the fluorogenic peptide pERTKR-MCA, as previously described [37]. In brief, cancer cells, and tumor tissues extracts were incubated with pERTKR-MCA (100 μM) during the indicated time periods in the presence of 25 mM Tris (pH 7.4), 25 mM methyl-ethane-sulfonic acid, and 2.5 mM CaCl2, at 37°C, and the fluorometric measurements were performed using a spectrofluorometer (Tecan Infinite® F200 PRO, Tecan Group Ltd. France).

### Proliferation assay

Tumor cells were plated in triplicate on 96 wells plate (2x10^3^/well) under serum free conditions for 24 h. The starved cells were then cultured during the indicated periods. Proliferation levels of cells was evaluated every two days using the Cell Titer96 non-radioactive cell proliferation assay kit (Promega) according to the manufacture's protocol.

### Soft agar assay

To assay anchorage-independent colony formation, HT-29/CTL, CT-26/CTL, HT-29/Spn4A or CT-26/Spn4A cells (4 × 10^3^ cells/well) were suspended in complete medium containing 0.8% agar and seeded in triplicate in six-well plates onto a basal layer of complete medium containing 3% agar. Complete medium was added every three days. After two weeks of cell growth, colonies >100 μm in diameter were counted using inverted microscopy and the results were represented as number of colonies formed, as previously described [5].

### Apoptosis assays and caspase-3 activity measurement

For apoptosis assays, tumor cells were grown to 70% confluency and cells were washed repeatedly to remove serum and then incubated for 6 h in media containing or not 5 mM H_2_O_2_ or 1 μM staurosporin. Cells were washed and stained with Phycoerythrin (PE)-labeled annexin V (AN) and 7-amino-actinomycin D (7AAD) using the PE Annexin V Apoptosis Detection Kit I (BD Pharmingen™) as instructed by the manufacturer. Cells were analyzed by flow cytometry (FACS CamptoII). The populations AN^−^/7AAD^−^, AN^+^/7AAD^−^, AN^−^/7AAD^+^, and AN^+^/7AAD^+^ that correspond to live cells, early apoptotic cells, necrotic cells and late apoptotic cells, respectively, were enumerated. Caspase-3 activity was determined in control and Spn4A-expressing cells following incubation for 6 h in media containing or not 5 mM H_2_O_2_ or 1 μM staurosporin, using the Caspase-3 Fluorescence assay Kit, as described by the manufacture (Cayman Chemical Company).

### Cell migration and invasion assays

Cell migration and invasion were determined using 24-well microchemotaxis chambers alone or precoated with 7.5 μg collagen type IV (Becton Dickinson Labware), respectively [5, 26]. Control tumor cells or cells expressing Spn4A cDNA were resuspended in serum-free media and loaded into upper chamber of each well. Cells were incubated at 37°C for 24 h, after which, the filters were fixed and stained with Diff-Quik (Medion Diagnostic). Cell migration and invasion were quantified by determination of the number of cells that migrated directly through the membrane toward the medium containing 10% serum that was used as a chemoattractant. Cells detected in each well were counted and the results were represented as (number of migrated cells/number of total cells) × 100%.

### Gelatin zymography

Zymography assay was done as previously described [5, 26], on serum-free conditioned media derived from control cells or cells stably transfected with Spn4A cDNA. SDS-PAGE gels were copolymerized with gelatin and samples were loaded onto gels without boiling. The gels were washed at room temperature in renaturing solution and in 10 mM of Tris-HCl (pH 8). For enzymatic reaction to take place, gels were incubated at 37°C in a solution of 50 mM of Tris-HCl (pH 8) containing 10 mM of CaCl_2_. The gels were then stained in Coomassie blue R250 solution and regions without staining were indicative of gelatin lysis.

### Tumorigenicity assay

Ethical approval for all animal studies was obtained from the Institutional Animal Care and Use Committee of the INSERM Institute in accordance with the National Advisory Committee for Laboratory Animal Research Guidelines licensed by the French Authority. Female 4- to 6-week-old nu/nu mice from Janvier Laboratories, housed in a pathogen-free facility, were used for all of the experiments. To assess the effect of PCs inhibition by Spn4A on tumor growth, 1x10^6^ HT-29, CT-26, HT-29/Spn4A, or CT-26/Spn4A cells were injected subcutaneously into nude mice, tumor formation was monitored every 2-3 days, and mice were sacrificed in the end of the experiments. Tumor volume was calculated as previously described [5, 6].

### Liver metastasis assay

Experimental liver metastases were generated by intrasplenic/portal injection of HT-29/CTL, HT-29/Spn4A, CT-26/CTL or CT-26/Spn4A cells, as previously described [6]. Because of high aggressiveness of CT-26 colon cancer cells, CT-26/CTL and CT-26/Spn4A cells-injected nude mice were sacrificed two weeks after the injection, whereas HT-29- and HT-29/Spn4A cells-injected mice were sacrificed four weeks later. Livers were removed and metastases were enumerated, without prior fixation.

### Immunohistochemical analyses

Developed tumors derived from injection of HT-29/CTL, HT-29/Spn4A, CT-26/CTL or CT-26/Spn4A cells, were analyzed for *in vivo* apoptosis using TumorTACS™ In Situ Apoptosis Detection Kit (Trivigen), as instructed by the manufacturer. The assay allows detection of chromosomal DNA fragments in the presence of deoxynucleotidyl transferase (TdT) that incorporated biotinylated nucleotides. The latter are visualised as dark brown precipitate through their biding to streptavidin-horseradish peroxydase and interaction with diaminobenzidine (DAB).

### Statistics

Unless otherwise indicated, Student's *t* test was used to determine the statistical significance of differences between the means of several experiments. A probability value less than 0.05 was considered to be statistically significant.

## SUPPLEMENTARY FIGURES



## SUPPLEMENTARY TABLES AND REFERENCES


