# Supplementary material for: Repression of liver colorectal metastasis by the serpin Spn4A a naturally occurring inhibitor of the constitutive secretory proprotein convertases
Source: Oncotarget. 2014 May 13;5(12):4195–210. doi: 10.18632/oncotarget.1966 (PMC4147316; doi:10.18632/oncotarget.1966)
Supplement: Supplementary file 1 [file oncotarget-05-4195-s001.pdf]

Repression of liver colorectal metastasis by the serpin Spn4A a naturally occurring inhibitor of the constitutive secretory proprotein convertases

Supplementary Material

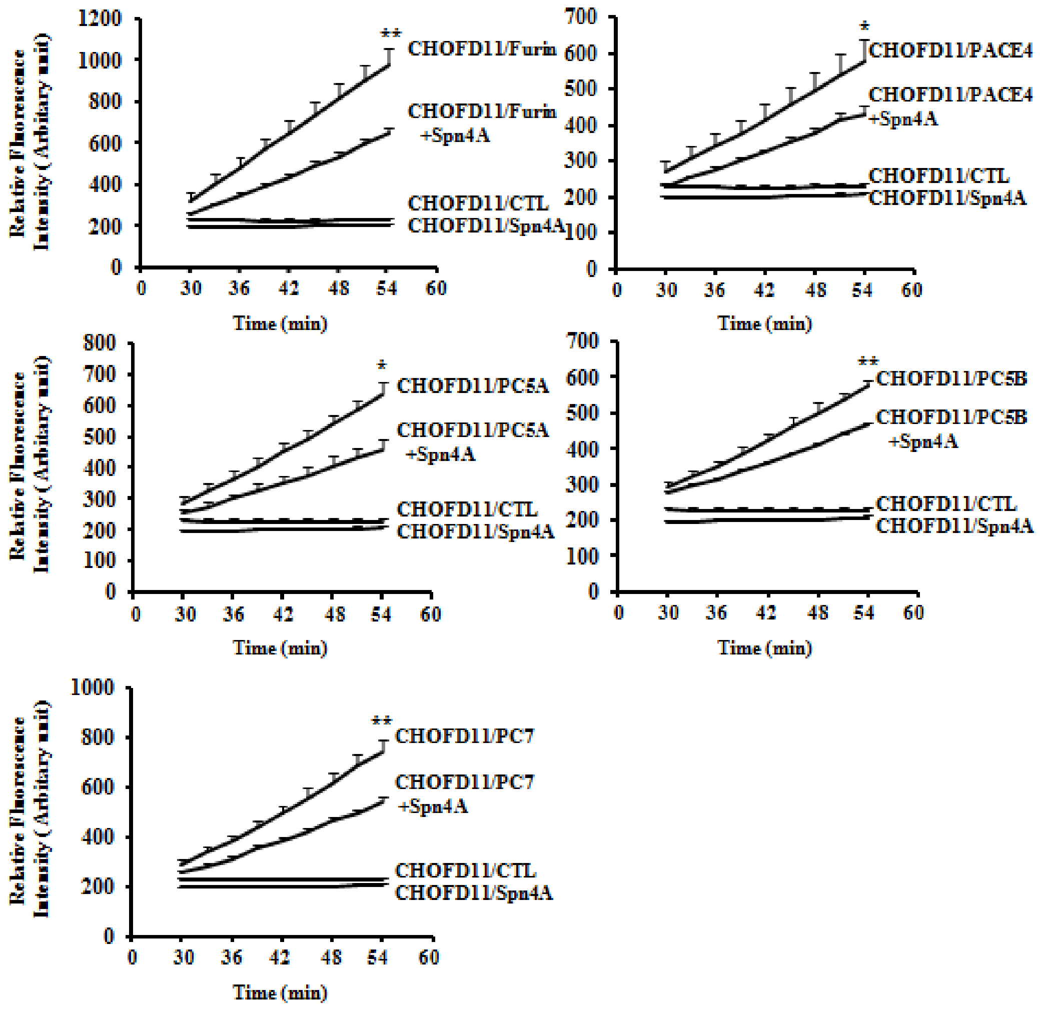

Figure 1: The effect of Spn4A on the activity of indicated PCs. PCs activity was assessed by evaluating their ability to digest the universal PCs substrate, the fluorogenic peptide pERTKR-MCA at the indicated time points in the presence of media derived from the deficient PCs cells CHOFD11, stably transfected with empty pIRES2-EGFP vector (CTL) or pIRES2-EGFP vector containing indicated PCs in the presence or absence of media derived from FD11 stably expressing Spn4A. Spn4 inhibited the activity of all PCs tested. Results are representative of three experiments and data are mean ± S.D performed in triplicate. *P < 0.05. ** P < 0.001.

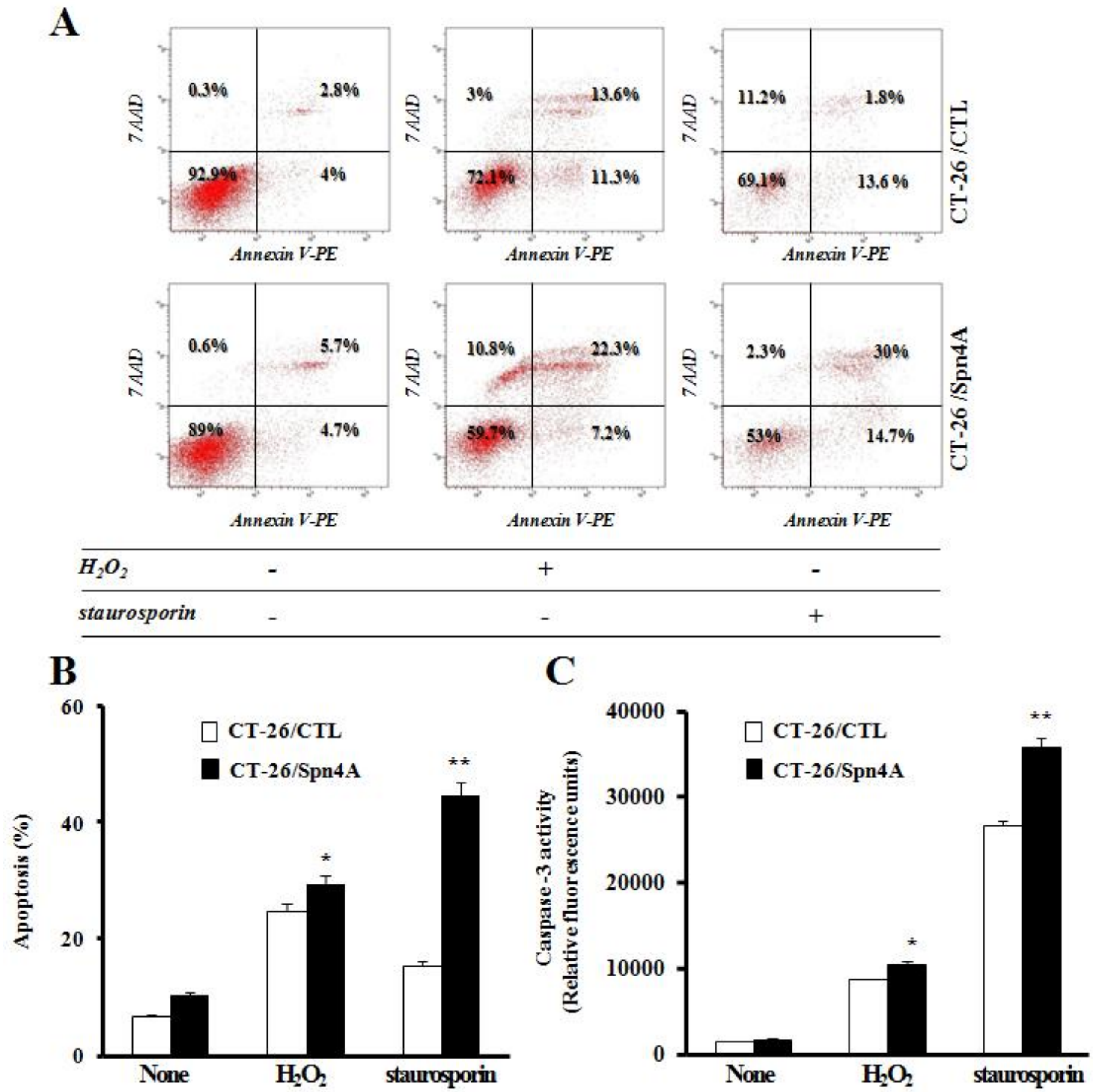

Figure 2. (A), **FACS scatter plots of CT-26/CTL and CT-26/Spn4A cells. (A)**, Cells were incubated for 6 h with H_2O_2 (5mM) or staurosporin (1mM) and double stained with annexin V and 7AAD. Fluorescence was detected using a fluorescence-activated cell sorter to analyze viable (negative for both dyes; lower left), early apoptotic (Annexin+/7AAD−, lower right), necrotic cells (Annexin-/7AAD+, upper left), and late apoptotic (Annexin+/7AAD+, upper right). **(B),** Percentages of apoptotic cells under these conditions are indicated. **(C),** tumor cells were incubated for 6 h with H_2O_2 (5mM) or staurosporin (1μM) and caspase-3 activity was evaluated using Caspase-3 Fluorescence assay Kit. Note that H_2O_2 and staurosporin caused an increased in the percentage of apoptotic cells that associated increased Caspase-3 activity. This effect was exacerbated; in cells-expressing Spn4A. Data shown represents the mean±SD from at least three independent experiments. *$P < 0.05$; **$P < 0.001$.

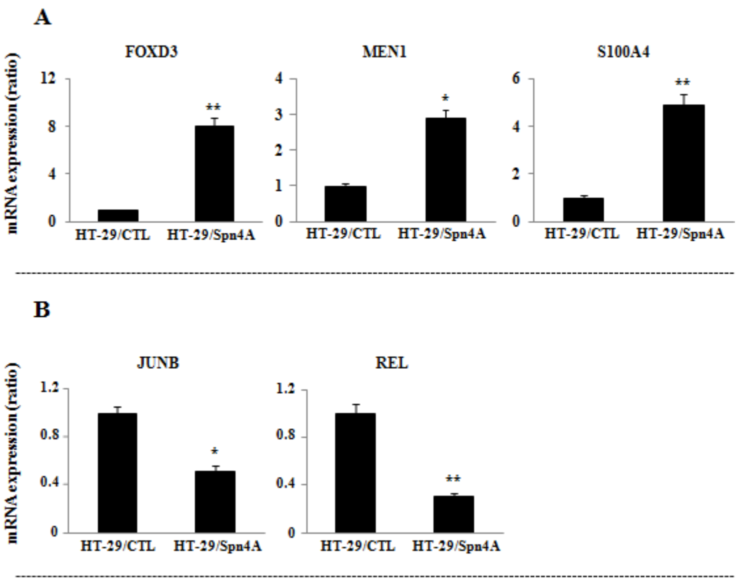

Figure 3: Induced human tumor suppressor genes by Spn4A and inhibited human oncogenes by Spn4A. Isolated total RNA was subject to RT[2] Profiler PCR array PAHS-502C for tumor suppressor genes **(A)** and oncogenes **(B)**. For each well, the results are expressed relative to the control cells transfected with empty vector (CTL) which was assigned a value of 1. Each value results are shown as means ± S.D. *P < 0.05; **P < 0.001.

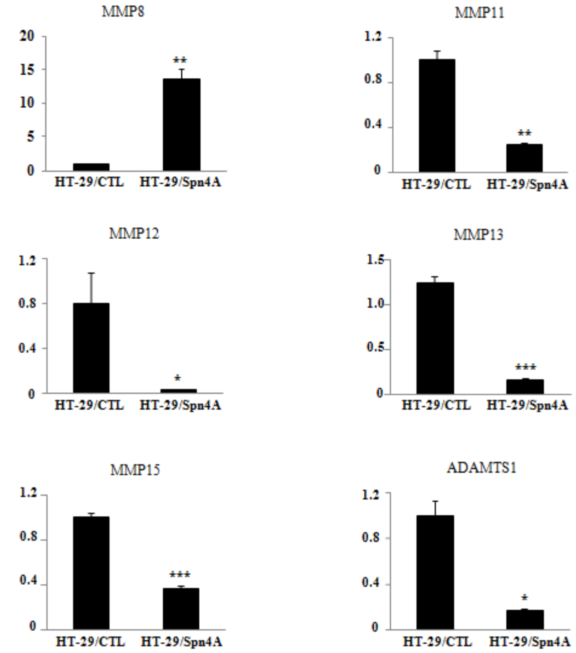

Figure 4: Effect of Spn4A on indicated MMPs expression. Isolated total RNA was subject to RT^2 Profiler PCR array PAHS-013C for MMPs. For each well, the results are expressed relative to the control cells transfected with empty vector (CTL) which was assigned a value of 1. Each value results are shown as means ± S.D. *$P < 0.05$; **$P < 0.001$. *** $P < 0.0001$.

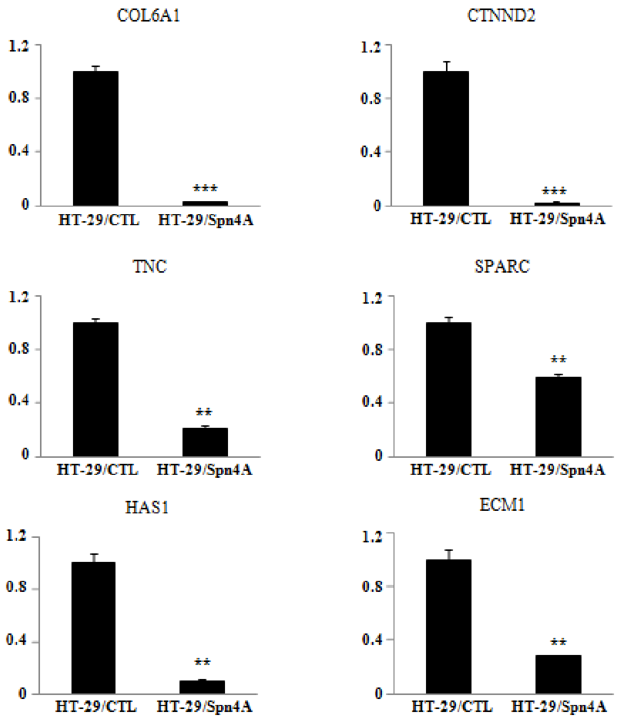

Figure 5: Inhibition of ECM constituents expression by Spn4A. Isolated total RNA was subject to RT^2 Profiler PCR array PAHS-013C for ECM. For each well, the results are expressed relative to the control cells transfected with empty vector which was assigned a value of 1. Each value results are shown as means ± S.D. *$P < 0.05$; **$P < 0.001$. *** $P < 0.0001$.

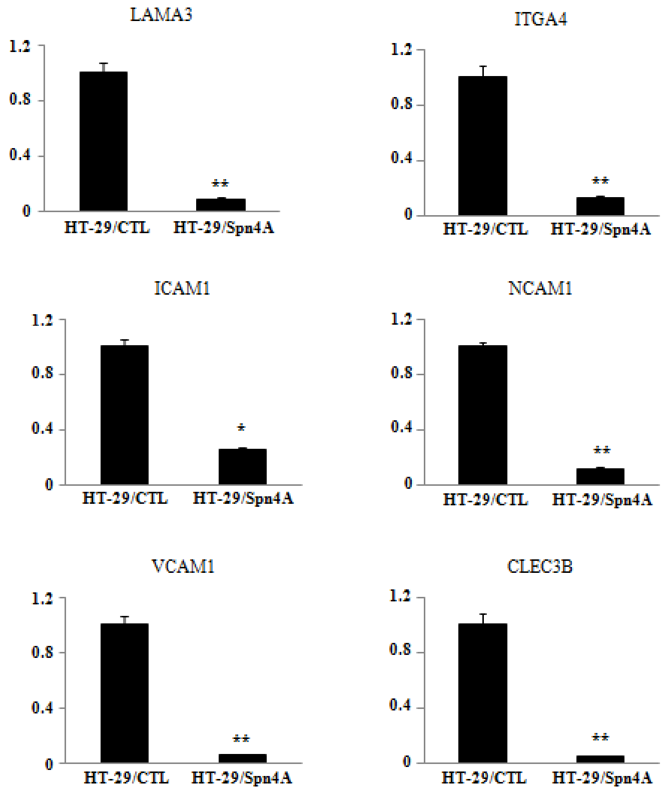

Figure 6. Inhibition of adhesion molecules expression by Spn4A. Isolated total RNA was subject to RT^2 Profiler PCR array PAHS-013C for adhesion molecules. For each well, the results are expressed relative to the control cells (CTL) transfected with empty vector which was assigned a value of 1. Each value results are shown as means ± S.D. *P < 0.05; **P < 0.001.
