# Supplementary material for: Repression of liver colorectal metastasis by the serpin Spn4A a naturally occurring inhibitor of the constitutive secretory proprotein convertases
Source: Oncotarget. 2014 May 13;5(12):4195–210. doi: 10.18632/oncotarget.1966 (PMC4147316; doi:10.18632/oncotarget.1966)
Supplement: Supplementary file 2 [file oncotarget-05-4195-s002.pdf]

Table-1: List of genes regulated by Spn4A in the colon carcinoma cells.

Gene Symbol	Gene banque reference	Name	Fonction	Fold regulation	Relevance in cancer and references
**ADAMTS1**	NM_006988	ADAM metallopeptidase with thrombospondin type 1 motif, 1	extramolecular Matrix Proteins: ECM Protease	-7.5	Involved in cell invasion (1) and tumor pregression (2)
**COL11A1**	NM_080629	Collagen, type XI, alpha 1	Extramolecular Matrix Proteins: collagens and ECM structural Constituants; protumoral propertises	+2.7	Involved in cell invasion (3)
**COL12A1**	NM_004370	Collagen, type XII, alpha 1	Extramolecular Matrix Proteins: collagens and ECM structural Constituants; protumoral propertises	+3.8	Marker of tumor invasion fronts (4)
**COL16A1**	NM_001856	Collagen, type XVI, alpha 1	Extramolecular Matrix Proteins: collagens and ECM structural Constituants; protumoral propertises	-1.8	Involved in cell invasiveness (5)
**CTNND2**	NM_001332	Catenin (cadherin-associated protein), delta 2 (neural plakophilin-related arm-repeat protein)	Extramolecular Matrix Proteins: collagens and ECM structural Constituants; protumoral propertises	-54.5	Involved in malignant progression and promotes cell invasion (6)
**ECM1**	NM_004425	Extracellular matrix protein 1	extramolecular Matrix Proteins protumoral and prometastatic propertises	-4.4	Implicated in cell proliferation, angiogenesis and differentiation (7)
**HAS1**	NM_001523	Hyaluronan synthase 1	extramolecular Matrix Proteins protumoral and prometastatic propertises	-13	Facilitate tumor progression by enhancing invasion, growth, angiogenesis and metastasis (8)
**ICAM1**	NM_000201	Intercellular adhesion molecule 1	Cell adhesion molecules: protumoral propertises	-4.8	Prometastatic effect (9)

**ITGA1**	NM_181501	Integrin, alpha 1	Cell-Matrix adhesion molecules: protumoral propertises	+3.6	Implicated in cell adhesion. Have a high metastatic abilities (10)
**ITGA4**	NM_000885	Integrin, alpha 4 (antigen CD49D, alpha 4 subunit of VLA-4 receptor)	Cell-Matrix adhesion molecules: protumoral propertises	-9.4	Involved in cell spreading and migration (11)
**LAMA3**	NM_000227	Laminin, alpha 3	extramolecular Matrix Proteins: basement membrane constituent, protumoral propertises	-13.5	Play a role in tumor progression and metastasis (12)
**MMP11**	NM_005940	Matrix metallopeptidase 11 (stromelysin 3)	extramolecular Matrix Proteins: ECM Protease	-5	Associated with tumor progression and poor prognosis (13)
**MMP12**	NM_002426	Matrix metallopeptidase 12 (macrophage elastase)	extramolecular Matrix Proteins: ECM Protease	no expression in Spn4A expressing cells	Overexpression correlate with presence of venous infiltration, high serum AFP level, early tumor recurrence and poor overall survival (14)
**MMP13**	NM_002427	Matrix metallopeptidase 13 (collagenase 3)	extramolecular Matrix Proteins: ECM Protease	-9.2	Frequently present and active in colorectal cancer. Its activity is associated with poorer survival (15)
**MMP15**	NM_002428	Matrix metallopeptidase 15 (membrane-inserted)	extramolecular Matrix Proteins: ECM Protease	-3.4	Seems to be up-regulated during tumorogenesis (16)
**MMP8**	NM_002424	Matrix metallopeptidase 8 (neutrophil collagenase)	extramolecular Matrix Proteins: ECM Protease	+11.1	Loss-of-function enhances the progression of melanoma (17)<br>Expression in breast cancer associated with diminished risk of lymph node metastasis (18)
**NCAM1**	NM_000615	Neural cell adhesion molecule 1	Cell adhesion molecules: protumoral propertises	-10.1	Has anti apoptotic properties, mediate cell-cell cohesion, enabling the formation of cell aggregates (19).

**SPARC**	NM_003118	Secreted protein, acidic, cysteine-rich (osteonectin)	Cell-Matrix adhesion molecules: anti-tumoral propertises	-2.1	Inhibits cell proliferation, spreading and migration (20)
**TIMP3**	NM_000362	TIMP metallopeptidase inhibitor 3	extramolecular Matrix Proteins: ECM Protease inhibitor	-12.8	Inhibites tumor growth and cell invasion (21)
**CLEC3B**	NM_003278	C-type lectin domain family 3, member B	Extramolecular Matrix Proteins: protumoral propertises	-117.3	Promote invasion, have a potential role in cell survival (22)
**TNC**	NM_002160	Tenascin C	Cell adhesion molecules: protumoral propertises	-5.8	Promote cancer cell growth, migration, adhesion, invasion and metastasis (23)
**VCAM1**	NM_001078	Vascular cell adhesion molecule 1	Cell adhesion molecules: protumoral propertises	-20.2	Implicated in cell adhesion and metastasis (24)
**BCL2**	NM_000633	B-cell CLL/lymphoma 2	tumor suppressor, anti apoptotic propertises	-3.9	Anti-apoptotic early expressed in colon carcinoma (25, 26)
**ESR1**	NM_000125	Estrogen receptor 1	both oncogene and tumor suppressor propertises	+2.3	Hypermethylation in colorectal cancer associated with risk of recurrence (27)
**FOXD3**	NM_012183	Forkhead box D3	tumor suppressor	+6.5	Down regule migration and invasion (28) Required for cell differentiation Negative (29) cell cycle regulator (30)
**JUNB**	NM_002229	Jun B proto-oncogene	oncogene	-2.3	Increase cell proliferation (31) Induce morphological and anchorage-independant cell growth consistent with a transformation like phenotype (32)
**MEN1**	NM_000244	Multiple endocrine neoplasia I	tumor suppressor	+2.4	Tumor suppressor Mutation leads to cell proliferation (33)

**MYB**	NM_005375	V-myb myeloblastosis viral oncogene homolog (avian)	both oncogene and tumor suppressor propertises	+8.1	Blok differentiation Regulator of stem and progenitor cells in born marrow (34)
**REL**	NM_002908	V-rel reticuloendotheliosis viral oncogene homolog (avian)	oncogene	-4	Overexpression contribute to inhibition of growth arrest and apoptosis of tumor cells (35)
**S100A4**	NM_002961	S100 calcium binding protein A4	tumor suppressor	+3.99	Interact with p53 tumor suppressor protein and induce apoptosis (36)
**TNF**	NM_000594	Tumor necrosis factor	both oncogene and tumor suppressor propertises, anti apoptotic propertises	+9.88	Expression is down regulate in high stage colorectal tumors Stimulate cell death/apoptosis **(37)**

Genes listed are those differentially regulated after expression of Spn4A in colon cancer cells HT-29 as compared to control HT-29 cells. The cut-off limit in the analysis was set to two-fold for both induced (+) and repressed (-) genes. The Gene Bank accession numbers are indicated.

Table 2: List of primers used for Real-Time PCR

Name	Sens	Sequence
H Furine	Forward	GCCCAGAATTGGACCACAGT
	Reverse	TCCCGATGTCTTTGGGCTC
	Probe	CAGCGGAAGTGCATCATCGACATCC
H PACE4	Forward	GACCTCAGTCTCTGCCCCC
	Reverse	TAACTGGCTGTTTGCTTCTAGAGC
	Probe	TGGTGGCGGGCATCATCGC
H PC5A	Forward	GGGAGCCATTTGCAAGGA
	Reverse	TTGGCACAGATTGTTCTTTTTCAC
	Probe	AAGAGTCCTGGGCGGAAGGAGGCT
H PC5B	Forward	CCAACCCAGGAAGACTGCA
	Reverse	GTTCGAAACACAGCGGCC
	Probe	ACCTGCCCCATGACAATTTTTGATG
H PC7	Forward	AGCATGGACTCGGATCCCA
	Reverse	CCCCCAGCATCGCACA
	Probe	CGGCTTCAATGACTGGACCTTCTCCA
M Furine	Forward	TGAGCCATTCGTATGGCTACG
	Reverse	TGCGCACCTCTAGCCGTT
	Probe	TGGTGGAACCCAAGGACATCGGC
M PACE4	Forward	CCGGACTTTATGCTGATGAAAGT
	Reverse	TCTCAGGTTCATCCACGCAC
	Probe	AGAGACTCTGCCTCAGGTGCCACCC
M PC5A+B	Forward	GGAAGAGGGAAGGTGTGTACAA
	Reverse	GCTGGAACAGTTCTTGAATCCT
	Probe	TGGACCACTCTTCAGAGGGTGGCT
M PC5B	Forward	GCTGCAGGATCGACGAAAG

	Reverse	GGACATGAAGCGCAGAGAGAT
	Probe	CCAGAATCAACCTTGTCACTCTTCTTGTAAAACCT
M PC7	Forward	GGGCCAGCACAATGACAACT
	Reverse	ATAAAAAGGCATCCGTCCCTC
	Probe	TGCCAACTCCATCTACACTGTCACCATAGG
M MMP2	Forward	GAGGACTATGACCGGGATAAGAAGT
	Reverse	GGGCACCTTCTGAATTTCCA
	Probe	TCCCGAGACCGCTATGTCCACTGTG
M MMP9	Forward	AAAGACCTGAAAACCTCCAACCT
	Reverse	GCCCGGGTGTAACCATAGC
	Probe	ACACCCAGCTGGCAGAGGCATACTTG
M TIMP1	Forward	AACTCGGACCTGGTCATAAGGG
	Reverse	TTAGTCATCTTGATCTTATAACGCTGGTA
	Probe	TTCCCCAGAAATCAACGAGACCACCTT
M TIMP2	Forward	TGCCCTGGGACACGCTTA
	Reverse	GGACAGCGAGTGATCTTGCA
	Probe	ATCACCCAGAAGAAGAGCCTGAACCACAG
M UPA	Forward	GGG-AAA-CAC-AAT-TAC-TGC-AGG-AA
	Reverse	CAT-TCT-TGG-ACA-AAC-TGC-CTT-AGG
M UPAR	Probe	CCT-GAC-AAC-CAG-AAG-CGA-CCC-TGG
	Forward	GAG-CTG-CCT-GGT-AGA-GGA-GTG-T
	Reverse	CAC-CTC-CAG-CTC-TCT-ATC-ATC-TTG
M PAI-1	Probe	CAG-GAC-TAC-CGT-GCT-TCG-GGA-ATG
	Forward	CTC-CAC-AGC-CTT-TGT-CAT-CTC-A
	Reverse	CAC-AAA-GAG-AAA-GGA-TCG-GTC-TAT-AA

(H : Human, M: Murine)

References

1. Tyan, S.W., Hsu, C.H., Peng, K.L., Chen, C.C., Kuo, W.H., Lee, E.Y., Shew, J.Y., Chang, K.J., Juan, L.J., and Lee, W.H. 2012. Breast cancer cells induce stromal fibroblasts to secrete ADAMTS1 for cancer invasion through an epigenetic change. *PLoS One* 7:e35128.
2. Hirano, T., Hirose, K., Sakurai, K., Makishima, M., Sasaki, K., and Amano, S.. Inhibition of tumor growth by antibody to ADAMTS1 in mouse xenografts of breast cancer. *Anticancer Res* 2011; 31:3839-3842.
3. Kim, H., Watkinson, J., Varadan, V., and Anastassiou, D. Multi-cancer computational analysis reveals invasion-associated variant of desmoplastic reaction involving INHBA, THBS2 and COL11A1. *BMC Med Genomics* 2010; 3:51.
4. Karagiannis, G.S., Petraki, C., Prassas, I., Saraon, P., Musrap, N., Dimitromanolakis, A., and Diamandis, E.P.. Proteomic signatures of the desmoplastic invasion front reveal collagen type XII as a marker of myofibroblastic differentiation during colorectal cancer metastasis. *Oncotarget* 2012; 3:267-285.
5. Bauer, R., Ratzinger, S., Wales, L., Bosserhoff, A., Senner, V., Grifka, J., and Grassel, S.. Inhibition of collagen XVI expression reduces glioma cell invasiveness. *Cell Physiol Biochem* 2011; 27:217-226.
6. Wang, M., Dong, Q., Zhang, D., and Wang, Y.. Expression of delta-catenin is associated with progression of human astrocytoma. *BMC Cancer* 2011; 11:514.
7. Wang, L., Yu, J., Ni, J., Xu, X.M., Wang, J., Ning, H., Pei, X.F., Chen, J., Yang, S., Underhill, C.B., et al.. Extracellular matrix protein 1 (ECM1) is over-expressed in malignant epithelial tumors. *Cancer Lett* 2003; 200:57-67.
8. Kim, H.R., Wheeler, M.A., Wilson, C.M., Iida, J., Eng, D., Simpson, M.A., McCarthy, J.B., and Bullard, K.M.. Hyaluronan facilitates invasion of colon carcinoma cells in vitro via interaction with CD44. *Cancer Res* 2004; 64:4569-4576.
9. Arteta, B., Lasuen, N., Lopategi, A., Sveinbjornsson, B., Smedsrod, B., and Vidal-Vanaclocha, F.. Colon carcinoma cell interaction with liver sinusoidal endothelium inhibits organ-specific antitumor immunity through interleukin-1-induced mannose receptor in mice. *Hepatology* 2010; 51:2172-2182.
10. Fukuda, K., Saikawa, Y., Yagi, H., Wada, N., Takahashi, T., and Kitagawa, Y.. Role of integrin alpha1 subunits in gastric cancer patients with peritoneal dissemination. *Mol Med Report* 2012; 5:336-340.
11. Weinlander, K., Naschberger, E., Lehmann, M.H., Tripal, P., Paster, W., Stockinger, H., Hohenadl, C., and Sturzl, M.. Guanylate binding protein-1 inhibits spreading and migration of endothelial cells through induction of integrin alpha4 expression. *FASEB J* 2008; 22:4168-4178.
12. Ii, M., Yamamoto, H., Taniguchi, H., Adachi, Y., Nakazawa, M., Ohashi, H., Tanuma, T., Sukawa, Y., Suzuki, H., Sasaki, S., et al.. Co-expression of laminin beta3 and gamma2 chains and epigenetic inactivation of laminin alpha3 chain in gastric cancer. *Int J Oncol* 2011; 39:593-599.
13. Barrasa, J.I., Olmo, N., Santiago-Gomez, A., Lecona, E., Anglard, P., Turnay, J., and Lizarbe, M.A.. Histone deacetylase inhibitors upregulate MMP11 gene expression through Sp1/Smad complexes in human colon adenocarcinoma cells. *Biochim Biophys Acta* 2012; 1823:570-581.
14. Ng, K.T., Qi, X., Kong, K.L., Cheung, B.Y., Lo, C.M., Poon, R.T., Fan, S.T., and Man, K.. Overexpression of matrix metalloproteinase-12 (MMP-12) correlates with poor prognosis of hepatocellular carcinoma. *Eur J Cancer* 2011; 47:2299-2305.
15. Leeman, M.F., McKay, J.A., and Murray, G.I.. Matrix metalloproteinase 13 activity is associated with poor prognosis in colorectal cancer. *J Clin Pathol* 2002; 55:758-762.
16. Sena, P., Mariani, F., Marzona, L., Benincasa, M., Ponz de Leon, M., Palumbo, C., and Roncucci, L. Matrix metalloproteinases 15 and 19 are stromal regulators of colorectal cancer development from the early stages. *Int J Oncol* 2012; 41:260-266.
17. Lopez-Otin, C., Palavalli, L.H., and Samuels, Y.. Protective roles of matrix metalloproteinases: from mouse models to human cancer. *Cell Cycle* 2009; 8:3657-3662.
18. Gutierrez-Fernandez, A., Fueyo, A., Folgueras, A.R., Garabaya, C., Pennington, C.J., Pilgrim, S., Edwards, D.R., Holliday, D.L., Jones, J.L., Span, P.N., et al.. Matrix metalloproteinase-8 functions as a metastasis suppressor through modulation of tumor cell adhesion and invasion. *Cancer Res* 2008; 68:2755-2763.
19. Wai Wong, C., Dye, D.E., and Coombe, D.R. 2012. The role of immunoglobulin superfamily cell adhesion molecules in cancer metastasis. *Int J Cell Biol* 2012:340296.
20. Nagaraju, G.P., and Sharma, D.. Anti-cancer role of SPARC, an inhibitor of adipogenesis. *Cancer Treat Rev* 2011; 37:559-566.

21. Tanaka, F., Sonoda, H., Okamoto, M., Mimori, K., Utsunomiya, T., Inoue, H., Hanai, T., and Mori, M.. TIMP-3 and Phosphatidylinositol 3-kinase genes were found to be related to the progression of colon cancer in a comparison of pneumoperitoneum and laparotomy in a murine model. *Surg Today* 2007; 37:220-225.
22. Westergaard, U.B., Andersen, M.H., Heegaard, C.W., Fedosov, S.N., and Petersen, T.E.. Tetranectin binds hepatocyte growth factor and tissue-type plasminogen activator. *Eur J Biochem* 2003; 270:1850-1854.
23. Chiquet-Ehrismann, R., and Chiquet, M. 2003. Tenascins: regulation and putative functions during pathological stress. *J Pathol* 200:488-499.
24. Yusuf-Makagiansar, H., Anderson, M.E., Yakovleva, T.V., Murray, J.S., and Siahaan, T.J.. Inhibition of LFA-1/ICAM-1 and VLA-4/VCAM-1 as a therapeutic approach to inflammation and autoimmune diseases. *Med Res Rev* 2002; 22:146-167.
25. Bronner, M.P., Culin, C., Reed, J.C., and Furth, E.E. The bcl-2 proto-oncogene and the gastrointestinal epithelial tumor progression model. *Am J Pathol* 1995; 146:20-26.
26. Huerta, S., Goulet, E.J., and Livingston, E.H.. Colon cancer and apoptosis. *Am J Surg* 2006; 191:517-526.
27. Woodson, K., Weisenberger, D.J., Campan, M., Laird, P.W., Tangrea, J., Johnson, L.L., Schatzkin, A., and Lanza, E.. Gene-specific methylation and subsequent risk of colorectal adenomas among participants of the polyp prevention trial. *Cancer Epidemiol Biomarkers Prev* 2005; 14:1219-1223.
28. Katiyar, P., and Aplin, A.E. FOXD3 regulates migration properties and Rnd3 expression in melanoma cells. *Mol Cancer Res* 2011; 9:545-552.
29. Mundell, N.A., Plank, J.L., LeGrone, A.W., Frist, A.Y., Zhu, L., Shin, M.K., Southard-Smith, E.M., and Labosky, P.A. Enteric nervous system specific deletion of Foxd3 disrupts glial cell differentiation and activates compensatory enteric progenitors. *Dev Biol* 2012; 363:373-387.
30. Abel, E.V., and Aplin, A.E. 2010. FOXD3 is a mutant B-RAF-regulated inhibitor of G(1)-S progression in melanoma cells. *Cancer Res* 2900; 70:2891-
31. Piechaczyk, M., and Farras, R. Regulation and function of JunB in cell proliferation. *Biochem Soc Trans* 2008, 36:864-867.
32. Leaner, V.D., Kinoshita, I., and Birrer, M.J. AP-1 complexes containing cJun and JunB cause cellular transformation of Rat1a fibroblasts and share transcriptional targets. *Oncogene* 2003; 22:5619-5629.
33. Eng, C., and Ponder, B.A.. The role of gene mutations in the genesis of familial cancers. *FASEB J* 1993; 7:910-919.
34. Ramsay, R.G., and Gonda, T.J.. MYB function in normal and cancer cells. *Nat Rev Cancer* 2008; 8:523-534.
35. Lu, H., Yang, X., Duggal, P., Allen, C.T., Yan, B., Cohen, J., Nottingham, L., Romano, R.A., Sinha, S., King, K.E., et al.. TNF-alpha promotes c-REL/DeltaNp63alpha interaction and TAp73 dissociation from key genes that mediate growth arrest and apoptosis in head and neck cancer. *Cancer Res* 2011; 71:6867-6877.
36. C, E.L.N., Grum-Schwensen, B., Mansouri, A., Grigorian, M., Santoni-Rugiu, E., Hansen, T., Kriajevska, M., Schafer, B.W., Heizmann, C.W., Lukanidin, E., et al. Cancer predisposition in mice deficient for the metastasis-associated Mts1(S100A4) gene. *Oncogene* 2004;.23:3670-3680.
37. Storkson, R.H., Aamodt, R., Vetvik, K.K., Pietilainen, K., Bukholm, G., Jonsdottir, K., Vollan, H.S., Sonerud, T., Luders, T., Jacobsen, M.B., et al.. mRNA expression of adipocytokines and glucocorticoid-related genes are associated with downregulation of E-cadherin mRNA in colorectal adenocarcinomas. *Int J Colorectal Dis* 2012; 8:1021-7. .
